# Supplementary material for: Dynalign II: common secondary structure prediction for RNA homologs with domain insertions
Source: Nucleic Acids Res. 2014 Nov 21;42(22):13939–48. doi: 10.1093/nar/gku1172 (PMC4267632; doi:10.1093/nar/gku1172)
Supplement: SUPPLEMENTARY DATA [file supp_gku1172_nar-02021-z-2014-File012.zip › manual/Overview/Building_Requirements.html]

RNAstructure Installation and Overview: Building Requirements


|  |  |  |
| --- | --- | --- |
|  | RNAstructure Installation and Overview Building Requirements | - Contents - Index |
| Compilers  Installation of the RNAstructure repository for Unix, Linux, or Mac OS X requires a current C++ compiler. By default, RNAstructure assumes a GNU g++ compiler, but there are alternative options that may be specified in RNAstructure/compiler.h.   ---   Compiler Flags  To compile the RNAstructure repository programs properly, a group of compiler variables must be selected in RNAstructure/compiler.h that is appropriate to the operating system. By default, RNAstructure assumes a Linux system. To change variable types to a different operating system, place a pound (#) sign next to each of the Linux variables, and remove the pound signs from the variables in the operating system section of your choice.   ---   DATAPATH Variable  In order to successfully use the data tables in the RNAstructure repository during calculations, an environment variable called DATAPATH must be set to the location of the data tables folder on the current machine.  For example, in BASH, this is accomplished with:  export DATAPATH=[directory in which RNAstructure resides]/RNAstructure/data\_tables/   ---   RNAstructure Java GUI: Software  To build the JAVA interface, the Sun JAVA JVM (Java Virtual Machine) version 1.6 or later, is required. The version of the JVM that comes from the JDK (Java Development Kit) is essential, because the headers in the "include" directory are necessary for successful integration of the interface with the back end. *Note that using other JVMs than the Sun JVM may cause aberrant program behavior and/or crashes.*   ---   RNAstructure Java GUI: Variables  The JAVA interface requires that two variables, INCLUDEPATH1 and INCLUDEPATH2, be set in compiler.h. These flags are required to find proper linking headers for the RNAstructure JAVA interface. On all systems, these paths point to the required native headers needed to interface JAVA with C++ code for the JAVA interface. These flags must be changed manually to coincide with the current machine before the JAVA interface can be run correctly, and are used in the RNAstructure\_java\_interface Makefile.  INCLUDEPATH1 must hold the directory which contains "jni.h."  INCLUDEPATH2 must hold the directory which contains "jni\_md.h."  Absolute paths must be used for both.   ``` For Fedora Linux, these two paths are normally similar to:     INCLUDEPATH1 = /usr/java/jdk1.x.x_x/include     INCLUDEPATH2 = /usr/java/jdk1.x.x_x/include/linux For Ubuntu Linux, these two paths are normally similar to:     INCLUDEPATH1 = /usr/lib/jvm/java-6-sun-1.x.x.x/include     INCLUDEPATH2 = /usr/lib/jvm/java-6-sun-1.x.x.x/include/linux For Mac, these two paths are usually the same, and similar to:     INCLUDEPATH1 = /System/Library/Frameworks/JavaVM.framework/Headers     For Mac, INCLUDEPATH2 should be defined as "", since it's not used. For Windows (Cygwin build), these two paths are normally similar to:     INCLUDEPATH1 = "C:\Program Files\Java\jdk1.x.x_x\include"     INCLUDEPATH2 = "C:\Program Files\Java\jdk1.x.x_x\include\win32"     For Windows, quotes may be needed if paths contain spaces. 							 ```   Note that the example paths given above are only meant to be a general guide to the paths' forms on different systems; the actual paths on any given system may differ from these.   ---   Building Multifind  To build Multifind, LIBSVM version 3.0 or later is required. Building Multifind also requires the variable INCLUDESVM to be set in compiler.h. It is the LIBSVM directory that contains svm.o and svm.h.   ---   Windows GUI or Text Interfaces  Building the Windows programs requires Microsoft Visual Studio, version 2010 or later, and the Intel C++ compiler, integrated into Visual Studio. The GUI solution for Visual Studio is found in the RNAstructure\_windows\_interface directory. Building the GUI requires Visual Studio Professional because of the use of MFC for building the Windows components. The text (command line) interface solution is found in the RNAstructure\_windows\_text\_interfaces directory. Each command line interface also has its own Visual Studio solution in the same directory as the interface.   ---    CUDA-enabled Software  Building partition-cuda requires a CUDA compiler, availble from NVIDIA:  https://developer.nvidia.com/cuda-downloads | | |
| Visit The Mathews Lab RNAstructure Page for updates and latest information. | | |
